# Localized amyloid-β oligomers in the lateral entorhinal cortex drives olfactory dysfunction through aberrant neuronal and network activity

**DOI:** 10.1186/s40478-026-02354-3

**Published:** 2026-06-23

**Authors:** Ting Pan, Jiaxing Fang, Xiao Wang, Yimeng Chen, Shan Li, Gaoxue Qiu, Changcheng Sun, Chao Liu, Anan Li, Fengjiao Chen

**Affiliations:** 1 Jiangsu Key Laboratory of Brain Disease and Bioinformation, Research Center for Biochemistry and Molecular Biology, Xuzhou Medical University, 209th Tongshan Road, Xuzhou, 221004 Jiangsu China; 2 Jiangsu Province Key Laboratory of Anesthesiology, Jiangsu Province Key Laboratory of Anesthesiology and Brain Science, NMPA Key Laboratory for Research and Evaluation of Narcotic and Psychotropic Drugs, School of Anesthesiology, Xuzhou Medical University, Xuzhou, China

**Keywords:** Lateral entorhinal cortex, Amyloid-β, Olfactory, AD‑related olfactory dysfunction

## Abstract

Olfactory dysfunction is one of the most common early features of Alzheimer’s disease (AD), yet its underlying neural mechanisms remains unclear. The lateral entorhinal cortex (LEC), a central node in the olfactory network, is among the earliest regions affected by AD pathology. However, the direct evidence supporting a causal link between LEC dysfunction and olfactory disorders in early AD is limited. In the present study, we explored whether and how LEC dysfunction contributes to early AD olfactory dysfunction by injecting amyloid-β_1−42_ (Aβ_1−42_) oligomers into the LEC to induce localized Aβ oligomers accumulation in this region. Our data demonstrate that localized Aβ oligomers in the LEC impaired odor detection and discrimination in mice. In vivo recordings revealed that Aβ oligomers increased baseline firing and odor-evoked activity in LEC neurons, while also enhancing odor-evoked beta oscillations. This hyperactive state corresponded with disrupted population-level odor decoding. Furthermore, deficits in synaptic structure and impaired synaptic transmission likely underlie the observed behavioral dysfunctions. Overall, these findings indicate that LEC dysfunction is linked to olfactory deficits in early AD, providing direct evidence for the involvement of this region in AD-related olfactory impairment.

**Supplementary Information:**

The online version contains supplementary material available at https://doi.org/10.1186/s40478-026-02354-3.

## Introduction

Alzheimer’s disease (AD) is a neurodegenerative disorder characterized by progressive cognitive decline and memory loss. Notably, olfactory system function is particularly vulnerable to AD. Olfactory dysfunction often emerges at the earliest clinical stages of the disease and has been proposed as a potential biomarker for assessing both the onset and progression of AD [20, 37, 44]. Consistent with these functional impairments, hallmark neuropathological features of AD, such as Aβ and Tau pathology, are prominently observed in brain regions involved in olfactory processing [12, 38, 41]. Olfactory dysfunction, therefore, is an important and early correlate of AD-related neuropathology.

Olfactory impairments in AD typically present as deficits in odor identification, detection, recognition, sensitivity and discrimination [19, 23]. Importantly, clinical studies report that deficits in odor identification and recognition are more pronounced than impairments in detection thresholds, which typically become evident only at advanced stages of the disease [26, 30, 45]. This pattern suggests that AD‑related olfactory dysfunction predominantly affects higher‑order cognitive processing, potentially reflecting a spatially selective progression of pathology within the olfactory network. Therefore, delineating the specific contributions of individual olfactory-related brain regions to AD-related olfactory decline is critical for improving early diagnosis and disease stratification.

The lateral entorhinal cortex (LEC) is a key olfactory hub, reciprocally connected with both the olfactory bulb and piriform cortex to form a core olfactory circuit [1, 3, 8, 15]. Functionally, the LEC modulates odor-evoked responses in the piriform cortex and plays an essential role in odor discrimination and odor-associated multimodal memory [3, 13, 18]. Notably, accumulating evidence indicates that the LEC is among the earliest brain regions affected during AD pathogenesis [9, 11, 32]. In AD mouse models, the LEC exhibits neuronal hyperexcitability and disrupted network oscillations at early disease stages [10, 28, 40]. However, the causal contribution of LEC dysfunction to olfactory deficits in AD remains largely unexplored.

In the present study, we investigated the role of the LEC in AD-related olfactory dysfunction by selectively injecting amyloid β_1−42_ (Aβ_1−42_) oligomers into the LEC of adult mice, since the accumulation of toxic Aβ oligomers is one of the earliest events in the molecular pathology of AD [31]. We demonstrated that localized Aβ_1−42_ oligomers accumulation in LEC induce impairments in olfactory sensitivity and discrimination. To elucidate the underlying neural mechanisms, we combined electrophysiology recordings with neuronal morphological analysis. Our results establish a direct link between LEC dysfunction and olfactory deficits, providing new insight into the neural basis of early sensory symptoms in AD.

## Materials and methods

### Preparation of Aβ_1−42_ oligomers

Aβ_1−42_ oligomers were generated followed the procedures described previously [31, 36]. Briefly, FITC labeled Aβ_1−42_ (GenicBio, Shanghai, China) was initially dissolved in 1,1,1,3,3,3-hexafluoro-2- propanol (HFIP, Sigma, USA) to a final concentration of 1 mM and incubated at room temperature for 1 h. The peptide solution was aliquoted and dried using a SpeedVac concentrator till traces of HFIP was removed under vacuum, which resulted in a monomerized Aβ film that was stored at − 80 °C.

For aggregation, the aliquoted peptide film was dissolved in dimethyl sulfoxide to 5 mM. The peptide solution was diluted directly into ice-cold phosphate-buffered saline (PBS, 1×) to a concentration of 450 µM. The mixture was then flicked to ensure complete dissolution of the film, sonicated for 5 min, and subsequently incubated at 4 °C for 16 h to generate the toxic oligomers. Following incubation, the solution was centrifuged at 13,000 × g for 15 min at 4 °C. The resulting supernatant was collected, diluted with PBS to a final concentration of 150 µM before subsequent stereotactic injections. To serve as a control, the FITC-labeled Aβ_42−1_ peptide (GenicBio, Shanghai, China) was also processed through the same aggregation protocol. Finally, The Aβ_1−42_ oligomers preparations were routinely characterized by Western blot using anti-Aβ antibody (_1−42_) (D9A3A) (Cell Signaling Technology, USA) and anti-FITC antibody (80003-1-RR, proteintech).

### Animals

Data for experiments were collected from male adult C57BL/6J strain mice over 8 weeks of age. Mice were housed in groups of four or five randomly under standard conditions (12-h light/dark cycle) with food and water available ad libitum. All procedures involving live mice were under the guidelines of the Institutional Animal Care and Use Committee guidelines of Xuzhou Medical University. The number and suffering of experimental animals were minimized.

### Stereotaxic injections

Mice were deeply anaesthetized by 1% pentobarbital sodium (90 mg/kg body weight, i.p.) and head-fixed in a stereotactic frame (RWD Instruments). The depth of anesthesia was confirmed by toe pinching. An incision was performed along the midline of the head and holes drilled in the skull at the desired coordinates. The prepared peptide solutions (600 nl) or specific virus solutions (200 nl) was injected into LEC (coordinates: anterior–posterior (AP), − 3.15 mm; medial–lateral (ML), ± 4.31 mm; dorso–ventral (DV), − 4.50 mm) using a pulled glass pipette connected to a pressure microinjector pump (The Stoelting Quintessential Injector; Stoelting Co.). The injection needle remained in place for approximately 10 min before being withdrawn to prevent fluid reflux. After surgery, mice were recovered from anesthesia on a heating pad and were then returned to their cages with ad libitum access to water and food. As postoperative care, carprofen (10 mg/kg) was given at 0 and 24 h after surgery to relieve pain and antibiotic (0.1 g/kg) was injected intraperitoneally for three consecutive days after surgery to prevent postoperative infection. Mice were given a minimum postoperative recovery period of one week before any subsequent procedures.

### TUNEL staining

TUNEL staining was performed according to standard protocols; detailed steps are described in the Supplementary Materials.

### Behavior testing

#### Go/no-go task

Water-restricted mice were trained in a freely moving behavioral paradigm to report odor detection using a go/no-go task as previously described [4, 35]. Initially, mice underwent a 2-day Go/Go (GG) training phase where they learned to lick a water port within a specific time window following presentation of either of two odors to receive a water reward. Subsequently, mice progressed to a Go/No-go (GNG) discrimination task, which included both easy and difficult versions. During the GNG task, mice were also presented by two different odors, but they were required to discriminate the two odors to receive the water reward. In the easy GNG task, the rewarded stimulus (S+) was 1% isoamyl acetate and the unrewarded stimulus (S−) was 1% 2-heptanone. The difficult version used binary mixtures of the same odorants at 6:4 (S+) and 4:6 (S−) ratios. Odors were presented randomly in all sessions. A lick to S+ within the response window was scored as a Hit and rewarded with water, while no lick to S+ was a Miss. For S− trials, licking was recorded as a False Alarm (FA) and not licking as a Correct Rejection (CR). The inter-trial interval was at least 10 s, and sessions typically consisted of 160–200 trials completed within 60 min per mouse. The performance of mice was evaluated daily throughout the training period. The overall correct rate was determined by dividing the sum of Hit and CR trials by the total number of trials (Hit + Miss + CR + FA).

#### Buried food pellet test

Olfactory function was assessed using the buried pellet test, which measures the time required for food-restricted mice to locate and uncover a food pellet hidden beneath bedding. Mice were food-deprived for 24 h prior to and during testing, with water available ad libitum. For each trial, a clean cage was filled with 3 cm of evenly distributed bedding. After a 10-minute habituation period in the test cage, the mouse was temporarily removed, and a 200 mg food pellet was buried 0.5 cm below the bedding surface. The mouse was then returned to the center of the cage and allowed up to 300 s to find the pellet. The latency to uncover the pellet was recorded; if the mouse failed to locate it within the allotted time, the trial was terminated and a maximum latency of 300 s was assigned. Following the test, the mouse was permitted to consume the pellet. As a control, a visible pellet test was conducted the following day under identical conditions, except that the pellet was placed on top of the bedding.

Additional methodological details pertaining to this section can be found in the Supplementary Materials. Mice that did not complete at least 90% of the behavioral trials or exhibited repetitive, non‑exploratory behavior were excluded.

### Surgery for microelectrode arrays implantation

Microelectrode arrays (16-channel, Kedou(Suzhou)Brain-Computer Technology Co. Ltd) were implanted into the LEC to record spikes and local field potential (LFP) as previously described [5, 42]. Mice were anesthetized with sodium pentobarbital (90 mg/kg, i.p.), secured in a stereotaxic apparatus, and body temperature was monitored and maintained by a heating pad. After applying eye ointment and shaving the scalp, the skull was exposed. A craniotomy was performed at coordinates targeting the right LEC (AP: −3.15 mm, ML: +4.31 mm). The microelectrode array was slowly implanted into the LEC region at a depth of about 4.4 mm, while neuronal firing was monitored to confirm optimal placement. Two other screw holes were drilled into the parietal bone for screws insertion which served as the ground. Finally, the implant was secured to the skull using dental acrylic, and a custom headplate was attached to facilitate head fixation during subsequent recordings. Postoperative care was provided as described above. After surgery, the mice were allowed a week to recover. The location of microelectrode arrays was histologically verified after all the experiments. Animals with electrodes placed outside the LEC were excluded from data analysis.

### In vivo electrophysiology recordings

Neural activity was recorded from awake, head-fixed mice freely moving on an air-lifted Styrofoam ball. To evoke responses, a set of eight odorants (isoamyl acetate, 2-heptanone, phenyl acetate, benzaldehyde, dimethylbutyric acid, n-heptanoic acid, n-pentanol, 2-pentanone, all purchased from Sinopharm Chemical Reagent Co.), each diluted to 1% (v/v) in mineral oil, was delivered via a nose-confronting port under a constant 1 L/min airstream. The stimulation protocol consisted of 2-second odor pulses presented 15 times per odor in pseudo-random order, with a 20-second inter-stimulus interval. Signals acquired via implanted microelectrodes were amplified and digitized by a 16-channel amplifier, with online referencing to a wire in the same brain region. Spike activity was band-pass filtered (300–5000 Hz) and sampled at 30 kHz, while LFPs were filtered (1–300 Hz) and sampled at 1 kHz. All neural data, synchronized with odor event markers, were recorded simultaneously using the NeuroStudio acquisition system for offline analysis.

### Offline spike sorting and unit data statistics

Neural spikes were sorted offline into single units using principal component analysis in Offline Sorter V4 (Plexon), following established methods. Peristimulus time histograms (PSTHs) were constructed using spike data from 4 s before to 6 s after odor onset, averaged across trials with a 100 ms bin width. The spontaneous firing rate was defined as the mean rate during the 2 s preceding odor stimulation, while the odor-evoked firing rate was calculated as the mean rate during the 2 s odor presentation. For each neuron-odor pair, a paired t-test was used to compare spontaneous and odor-evoked firing rates across trials. Neuronal responses were classified as excitatory, inhibitory, or non-significant based on the statistical p-value and the direction of firing rate change, with the response magnitude quantified by the normalized mean firing rate (MFR): (odor-evoked firing rate – spontaneous firing rate) / spontaneous firing rate.

### LFP signals analysis

Raw LFP signals were band-pass filtered and decomposed into four standard frequency bands: theta (2–12 Hz), beta (15–35 Hz), low gamma (36–65 Hz), and high gamma (66–95 Hz). Analysis was concentrated on the beta, low gamma, and high gamma bands. For these frequency ranges, data segments from 4 s before to 6 s after odor onset were extracted. Each trial was normalized to its pre-stimulus baseline (the 2 s period preceding odor onset), and trial-averaged responses were subsequently computed for each odor. The 50 Hz noise component within the low gamma range was corrected by interpolating the power values from the neighboring frequency points.

### Odor decoding analysis

Odor decoding was performed using population-level neural activity analyzed in Python 3.7.4. We implemented a linear support vector machine (SVM) classifier on data across all recording sessions. The input features for the classifier were single-trial response vectors of firing rates, constructed by concatenating non-overlapping 100-ms bins from specific post-stimulus periods as shown in Fig. 5A. Decoding accuracy was quantified via 10-fold cross-validation. As a control, identical analysis was applied to neural activity during the 2-second pre-odor baseline period to establish a chance-level performance baseline.

### Slice preparation and patch-clamp recordings

To prepare LEC slices, mice were deeply anesthetized and transcardially perfused with ice-cold, carbogen-saturated (95% O_2_/5% CO_2_) dissection solution containing (in mmol): 234 sucrose, 11 glucose, 24 NaHCO_3_, 2.5 KCl, 1.25 NaH_2_PO_4_, 0.5 CaCl_2_, and 10 MgSO_4_. Coronal brain slices (250 μm) containing the LEC region were prepared using a vibratome (Leica VT 1200 S). Slices were subsequently incubated in oxygenated artificial cerebrospinal fluid (ACSF) containing (in mmol): 126 NaCl, 26 NaHCO_3_, 2.5 KCl, 1.25 NaH_2_PO_4_, 2 CaCl_2_, 2 MgCl_2_, and 10 glucose. Incubation was performed at 37 °C for 60 min, followed by maintenance at 25 °C until electrophysiological recording.

For in vitro electrophysiology recordings, slices were continuously perfused with oxygenated ACSF (2 mL/min) in the recording chamber and visualized under infrared differential interference contrast microscopy using an upright microscope (Nikon ECLIPSE FN1) equipped with a 60× water-immersion objective (Zeiss Examiner.A1). LEC was identified based on its anatomical position relative to the rhinal fissure. Under epifluorescence illumination, regions exhibiting robust oligomeric fluorescence expression were selected for recording. Recordings were primarily targeted at neurons located in layers II/III of the LEC.

In current-clamp mode, recording pipettes were pulled from borosilicate glass capillaries (Sutter) to have resistances of 6–8MΩ and then filled with ACSF for action potentials (APs) recording in cell-attached mode. For AP recording in whole cell mode, pipettes were filled with a solution containing (in mM): 135 K-gluconate, 5 KCl, 0.5 CaCl_2_, 10 N-(2-hydroxyethyl) piperazine-N’-(2-ethanesulfonic acid) (HEPES), 2 Mg-ATP, 0.1 GTP, and 5 ethylene glycol-bis (2-aminoethylether)-N, N,N’,N’-tetraacetic acid (EGTA), 300 mOsm (pH adjusted to 7.3 with KOH). To evoke AP firing, 400-ms step currents at five intensities (50, 80, 110, 140, 170) were injected into cells with an intertrial interval of 30 s. The membrane potential at which phase plot slope exceeded 10 mV/ms was denoted threshold.

In voltage-clamp mode, excitatory and inhibitory postsynaptic currents (EPSCs/IPSCs) were recorded as previously described [5, 42]. To isolate glutamatergic currents, GABA A receptors were blocked with bicuculline (10 µM). To isolate GABAergic currents, α-amino-3-hydroxy-5-methyl-4-isoxazolepropionic acid (AMPA)/kainite receptors were blocked with 6-Cyano-7-nitroquinoxaline-2, 3-dione (CNQX, 10 µM), N-methyl-D-aspartate (NMDA) receptors were blocked with DL-2- amino-5-phosphonovaleric acid (APV, 50 µM). TTX (1 µM) was also included during the recording of miniature EPSCs/IPSCs (mEPSCs/mIPSCs).

Electrophysiological data were collected by a patch-clamp amplifier (Multiclamp 700B, Molecular Devices) and a digital-to-analog converter (Digidata 1440 A, Axon Instrument). Signals were sampled at 10 kHz and voltage signals were filtered at 2 kHz. Offline data analysis was performed in Clampfit 10.2 (Molecular Devices).

### Neuronal sparse labeling assay

To evaluate the effects of Aβ_1−42_ oligomers on dendritic density in LEC neurons, sparse neuronal labeling was performed. A mixture of 100 nL sparse-labeling AAV (NCSPYFP-2E5, Brain Case) and either Aβ_42−1_ or Aβ_1−42_ oligomers was injected into the LEC. After a 4-week expression period, brains were processed for histology, and coronal Sect.  (50 μm) containing the LEC were prepared. YFP-labeled neurons in LEC layers II/III were randomly selected and imaged using a confocal microscope (Zeiss, LSM710) with z-stack acquisition. Dendritic morphology was reconstructed from maximum intensity projections using ImageJ software. Dendritic complexity was further evaluated using Sholl analysis, with all quantitative analyses performed in a double-blind manner.

### Transmission electron microscopy (TEM)

To evaluate synaptic ultrastructure, LEC tissues from anesthetized mice were collected, trimmed into 1 mm³ cubes, and fixed overnight in cold glutaraldehyde. Subsequently, tissues were post-fixed in 1% osmium tetroxide, stained with 2% uranyl acetate, and dehydrated through a graded series of ethanol and acetone before epoxy resin embedding. Ultrathin Sect.  (70 nm) were prepared, counterstained with uranyl acetate and lead citrate, and imaged under a Tecnai G2 Spirit TWIN electron microscope. Postsynaptic density dimensions were quantified using ImageJ software.

### Statistical analysis

Animals were randomly grouped, and data collection and analysis were conducted blinded to the group identity. Statistical evaluations were carried out using MATLAB 2020a and GraphPad Prism 8.0. Normality of residuals was tested by the Anderson–Darling test along with Q-Q plots. For normally distributed data, comparisons between two groups utilized the t-test, and multi-group comparisons employed two-way ANOVA followed by Tukey’s post hoc test where appropriate. For non-normally distributed data were analyzed using the Wilcoxon rank-sum test (two groups) or corresponding non-parametric alternatives for multiple groups. For some datasets with hierarchical structure, nested t‑tests with animal as the experimental unit were also performed to account for within‑animal clustering. Additional analyses included the two-sample Kolmogorov–Smirnov test for continuous distributions and the chi-square test for proportional data. All results are expressed as mean ± SEM, with specific statistical tests indicated in the relevant results sections. Sample size was not determined by a prospective power analysis. The number of animals per group was chosen based on our previous work [5, 6, 16] and is indicated in the figure legends.

## Results

### Aβ oligomers restricted to the LEC impair olfactory sensitivity and odor discrimination

**Fig. 1:**
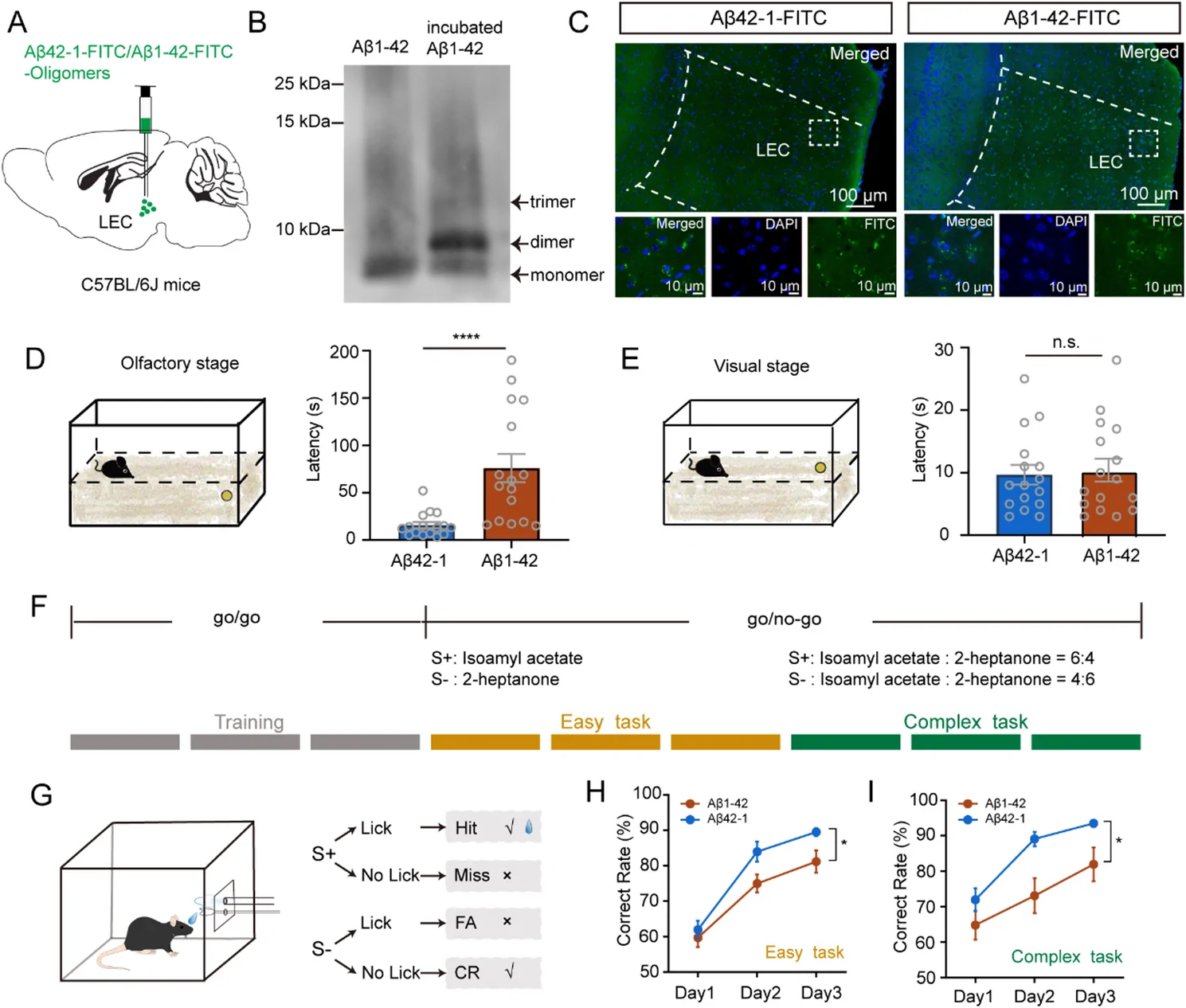
Aβ oligomers restricted to the LEC disrupts both odor detection and discrimination. **A** Schematic of the Aβ peptide injection protocol for generating mouse models with LEC‑restricted Aβ oligomer accumulation or control treatments. **B** Western blot analysis of Aβ_1−42_ monomer (left lane), and Aβ_1−42_ oligomer preparations (right lane) using anti-Aβ_1−42_ antibody after SDS-PAGE. The oligomer sample shows bands corresponding to monomers, dimers, and trimers. **C** Representative images showing the distribution of Aβ_42−1_ peptide and Aβ_1−42_ oligomers in the LEC at 4 weeks after viral injection. **D** Schematic of the buried pellet test (left) and the latency to locate a food pellet in Aβ_42−1_ versus Aβ_1−42_ mice (*n* = 16 mice per group). **E** Schematic of the visible pellet test (left) and the comparison of the time to find the food pellets between groups (right). **F–G** Schematics of the olfactory discrimination task (**F**) and the behavioral paradigm (**G**). **H–I** Odor-discrimination performance during the easy task (**H**) and the difficult task (**I**). Aβ_42−1_, *n* = 14 mice; Aβ_1−42_, *n* = 16 mice. *p < 0.05; ****p < 0.001

To determine whether dysfunction of the lateral entorhinal cortex (LEC) contributes to olfactory deficits associated with AD, we bilaterally injected oligomeric Aβ_1−42_ into the LEC of adult C57BL/6J mice to induce region-specific Aβ oligomer accumulation. To achieve focal and acute delivery of oligomers to the LEC, we injected 90 pmol (150 µM, 600 nL) of Aβ_1–42_ oligomers into the unilateral LEC. Although this dose is slightly higher than the endogenous level [40], it ensures the reliability and reproducibility of our experimental results, and is consistent with other studies in the field that used exogenous Aβ oligomer injection [36]. Control mice received bilateral LEC injections of the reverse peptide Aβ_42−1_ (Fig. 1A). Prior to in vivo experiments, oligomeric Aβ_1−42_ structures were characterized by Western blot analysis. As shown in Fig. 1B and Fig. S1A, Aβ_1–42_ preparations contained a mixture of monomers, dimers, and trimers, in contrast to the Aβ_42−1_ control, confirming the successful generation of oligomeric Aβ species. To assess the spatial distribution and persistence of Aβ oligomers, fluorescence imaging was performed on brain sections containing the LEC. FITC-labeled Aβ peptides were largely confined to the LEC at both 1 week (Fig. S1B) and 4 weeks post-injection (Fig. 1C), indicating stable and localized Aβ deposition. Moreover, Aβ oligomers were broadly distributed along the anteroposterior axis within the LEC (Fig. S1C). Accordingly, all subsequent experiments were conducted within 4 weeks after injection.

We first examined whether LEC-restricted Aβ oligomer accumulation induced neuronal apoptosis. Terminal deoxynucleotidyl transferase-mediated dUTP nick-end labeling (TUNEL) revealed very few TUNEL-positive cells in the LEC of either Aβ_42−1_ or Aβ_1–42_-treated mice (Fig. S2). We next assessed whether localized Aβ oligomer accumulation in the LEC impaired olfactory function. Olfactory sensitivity was first evaluated using the buried food pellet test. Mice injected with Aβ_1–42_ oligomers exhibited significantly longer latencies to locate the buried food pellet compared with control mice (Fig. 1D: Mann-Whitney test, *p* = 1.39 × 10⁻^5^, Mann-Whitney U = 22). In contrast, no difference was observed between groups when the food pellet was placed on the surface of the bedding (Fig. 1E: Mann-Whitney test, *p* = 0.93, Mann-Whitney U = 125.5), indicating preserved motivation and motor ability. To further examine higher-order olfactory processing, odor discrimination performance was assessed using a go/no-go behavioral paradigm with both easy and difficult odor pairs. Trial outcomes were classified as hit, CR, FA, or miss (Fig. 1F, G), and performance was quantified as the percentage of correct responses (hits + CRs). Compared with control mice, Aβ₁₋₄₂-treated mice showed significantly impaired discrimination performance for both easy (Fig. 1H: two-way ANOVA, *p* = 0.0466, *F*_(1,28)_ = 4.33) and difficult (Fig. 1I: two-way ANOVA, *p* = 0.0266, *F*_(1,28)_ = 5.48) odor pairs.

In addition to its role in olfactory processing, the LEC has been implicated in processing contextual information, including object and novelty recognition [27, 34]. We therefore assessed whether mice injected with Aβ_1−42_ oligomers exhibited deficits in a novel object recognition test (Fig. S3A). The results showed that Aβ₁₋₄₂-treated mice exhibited a trend toward reduced exploration of the novel object, reflected by a modest decrease in preference index compared with control mice (Fig. S3B: unpaired t test, *p* = 0.0674, *t*_(33)_ = 1.89). This mild impairment may reflect the strong dependence of LEC-mediated object recognition on hippocampal circuitry, which appears largely preserved in this model. Consistent with this interpretation, spatial working memory assessed using the Y-maze test was intact in Aβ_1–42_-treated mice, indicating preserved medial entorhinal cortex (MEC)-related function (Fig. S3C–D: unpaired t test, *p* = 0.884, *t*_(28)_ = 0.147). Finally, the locomotor activity in the open field remained comparable between the two groups (Fig. S3E–G, F: Mann-Whitney test, Mann-Whitney U = 87, *p* = 0.305; G: unpaired t test, *t*_(28)_ = 0.903, *p* = 0.374). Taken together, these results demonstrate that Aβ oligomers restricted to the LEC drive olfactory deficits in mice, highlighting a critical role of the LEC to AD-associated olfactory impairment.

### LEC-restricted Aβ oligomers enhances spontaneous firing and the odor-evoked responses of LEC neurons in awake mice

**Fig. 2:**
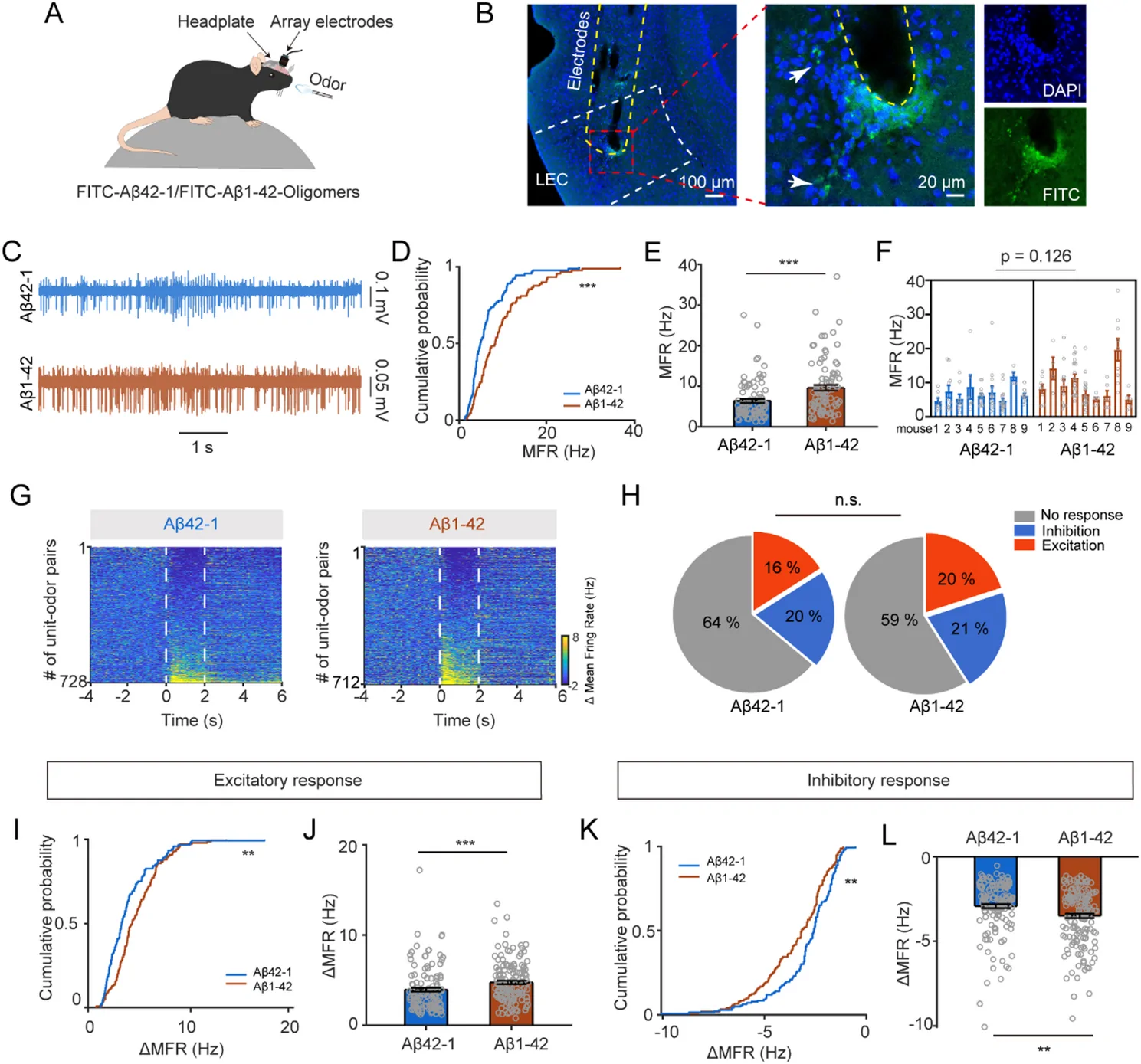
Localized Aβ oligomers enhanced the spontaneous firing and odor‑evoked responses in LEC neurons. **A** Schema of the experimental paradigm. Array microelectrodes were lowered into layer II/III of LEC to record the neuronal spiking. **B** Representative images from a single mouse show the microelectrode placement (left) and, at higher magnification (right), FITC‑labeled Aβ oligomers (arrows). **C** Representative raw spike traces from Aβ_42−1_ and Aβ_1−42_ mice. **D** Cumulative probability distribution of the baseline mean firing rate (MFR) for all recorded neurons. A total of 89 cells were identified from 9 Aβ_42‑1_ mice and 91 cells from 9 Aβ_1–42_ mice. **E**,** F** Comparison of spontaneous firing rates of LEC neurons between groups at single-unit level (**E**) and at the animal level (**F**), respectively. The animal‑level analysis was performed using nested t‑test with mouse as the experimental unit. **G** Heat maps of ΔMFR across all unit–odor pairs recorded from two group mice. **H** Comparison of the distribution of excitatory and inhibitory odor responses in two groups. **I**,** J** Aβ_1–42_ oligomers injection enhanced the excitatory odor responses. *n* = 120 unit–odor pairs for the Aβ_42−1_ group and *n* = 140 unit–odor pairs for Aβ_1–42_ group. **K**,** L** Inhibitory odor responses were augmented upon Aβ_1–42_ oligomers injection in LEC. *n* = 143 and *n* = 154 unit–odor pairs for the Aβ_42−1_ and Aβ_1–42_ groups, respectively. **p < 0.01; ***p < 0.001; n.s., not significant

To elucidate how Aβ_1−42_ oligomers impair olfaction, we recorded LEC neuronal activity in vivo from awake, head-fixed mice injected with either Aβ_42−1_ or Aβ_1−42_ oligomers (Fig. 2A). The location of microelectrodes was confirmed after recording (Fig. 2B). Representative firing traces of LEC neurons from one mouse per group are shown in Fig. 2C, and well-isolated single units were reliably identified through spike sorting (Fig. S4A). In total, we recorded 91 single units from control mice and 89 single units from Aβ_1−42_-treated mice (*n* = 9 mice per group). The distributions of spontaneous firing rates of all the recorded neurons are shown in Fig. 2D., Compared with controls, LEC neurons in the Aβ_1−42_ group exhibited significantly elevated spontaneous firing rates when analyzed at the single-unit level (Fig. 2D: two sample K-S test, *p* = 8.64 × 10^−4^; Fig. 2E: Wilcoxon rank sum test, *p* = 1.08 × 10^−4^). To account for clustering within animals, we performed a nested analysis to further evaluate the effect of Aβ oligomers on neuronal spontaneous firing. A possible enhancing effect was observed, though it did not reach statistical significance at the animal level (Fig. 2F: nested t test, *t*_(16)_ = 1.61, *p* = 0.126), likely due to between‑animal heterogeneity and limited statistical power. Taken together, these results suggest that local application of Aβ oligomers may enhance the ongoing baseline activity of LEC neurons.

Next, we examined how LEC neurons in control and Aβ_1–42_ mice responded to various odor stimuli in vivo. Consistent with previous studies, passive odor presentation elicited heterogeneous neuronal responses, including excitatory responses, inhibitory responses, and non-responsive units (Fig. S4B). Changes in mean firing rate (ΔMFR) during odor delivery for all recorded units are shown in Fig. 2G. The overall distribution of response types did not differ significantly between control and Aβ_1−42_ groups (Fig. 2H: Chi-square test, *p* = 0.118). Despite the preserved proportion of responsive neurons, the magnitude of odor-evoked responses was significantly enhanced in the Aβ_1−42_ group. Specifically, neurons exhibiting excitatory responses showed significantly larger increases in firing rate compared with controls (Fig. 2I: two sample K-S test, *p* = 1.39 × 10^−3^; Fig. 2J: Wilcoxon rank sum test, *p* = 3.92 × 10^−4^). Similarly, the magnitude of odor-evoked inhibitory responses was also significantly increased in Aβ_1−42_-treated mice (Fig. 2K: two sample K-S test, *p* = 2.03 × 10^−3^; Fig. 2L: Wilcoxon rank sum test, *p* = 1.39 × 10^−3^). Together, these results demonstrate that LEC‑restricted Aβ oligomers enhances both spontaneous firing and the magnitude of odor‑driven excitatory and inhibitory responses in LEC neurons.

### LEC-restricted Aβ oligomers amplifies local beta oscillatory responses to odors

**Fig. 3:**
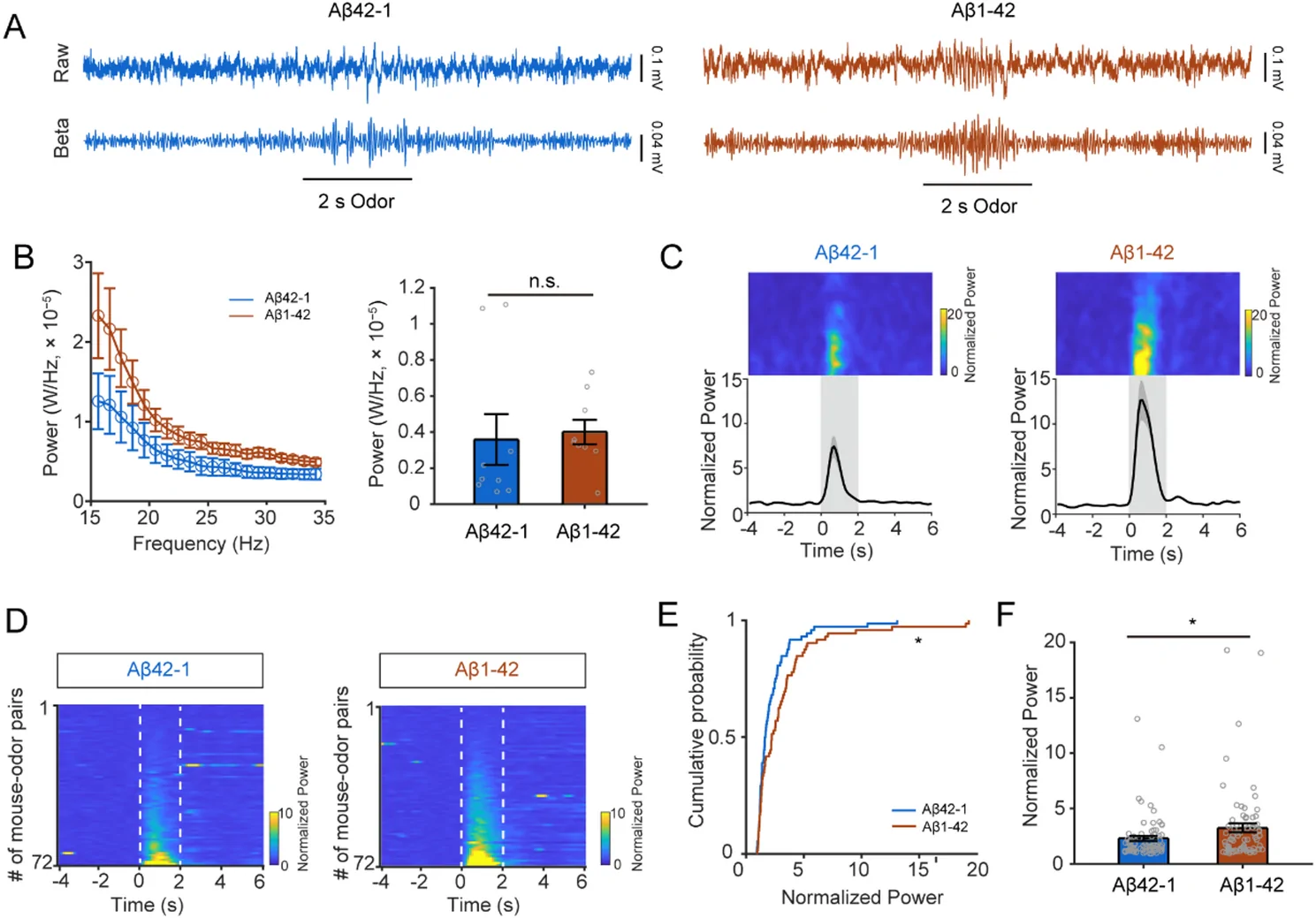
LEC‑restricted Aβ oligomers enhances the odor-evoked beta oscillatory responses. **A** Examples of raw LFP traces (top) and filtered beta-band oscillations (bottom) recorded in vivo from representative Aβ_42−1_ and Aβ_1–42_ mice. **B** Average power spectrum (left) and the statistical analysis (right) of the ongoing beta oscillations between groups. **C** Example power spectra (top) and trial-averaged normalized traces (bottom) of beta oscillatory responses elicited by odor delivery (gray boxes). **D** Odor-evoked beta oscillatory dynamics across all recorded mice. Cumulative probability (**E**) and group statistics (**F**) of normalized odor-evoked beta power (*n* = 72 odor–mouse pair per group). *p < 0.05; n.s., not significant

While single-unit spiking captures the activity of individual neurons, LFPs reflect the synchronized activity of neuronal ensembles and are proposed to be important for cell assembly synchronization and plasticity [2, 24]. First, we focused on the beta frequency range (15–35 Hz), which is thought to be robustly induced by odors, leading to pronounced oscillations. Consistent with previous findings [39], odor stimulation reliably evoked prominent oscillatory activity in both raw LFP traces and the filtered beta band signals in the LEC (Fig. 3A). We therefore asked whether LEC-restricted Aβ oligomers influences ongoing and odor-evoked beta oscillations. Baseline beta oscillatory activity did not differ significantly between Aβ_42−1_ and Aβ_1−42_-treated mice (Fig. 3B, right: Wilcoxon rank sum test, *p* = 0.190). In contrast, representative recordings revealed a marked increase in the amplitude of odor-evoked beta oscillations in Aβ_1−42_-treated mice compared with controls (Fig. 3C). Changes in beta oscillatory power during odor presentation across all recorded animals are summarized in Fig. 3D. When we compared the responses across different mice and odors, we found that the restricted Aβ_1−42_ oligomers in the LEC significantly increased the amplitude of the odor-evoked beta response (Fig. 3E: two-sample K-S test, *p* = 0.0299; Fig. 3F: Wilcoxon rank sum test, *p* = 0.0414). Overall, the Aβ oligomer accumulation enhanced beta oscillation dynamics in the LEC during odor stimulation.

Subsequently, we analyzed low gamma and high gamma oscillations, respectively. For baseline gamma oscillations, we found that Aβ oligomers tended to increase both the low gamma (Fig. S5A: right, Wilcoxon rank sum test, *p* = 0.161) and high gamma (Fig. S5B: right, Wilcoxon rank sum test, *p* = 0.0939) oscillations power, but the difference was not statistically significant. Moreover, gamma oscillations lacked a robust response to odor stimulation; only a minority of unit–odor pairs exhibited any response (Fig. S5C, D). Additionally, Aβ oligomers had no significant effect on either low gamma (Fig. S5E: two-sample K-S test, *p* = 0.0759; Fig. S5F: Wilcoxon rank sum test, *p* = 0.679) or high gamma (Fig. S5G: two-sample K-S test, *p* = 0.0537; Fig. S5H: Wilcoxon rank sum test, *p* = 0.125) oscillatory responses during odor delivery.

### Aβ oligomers disrupts the odor-decoding performance of the LEC neuronal populations

**Fig. 4:**
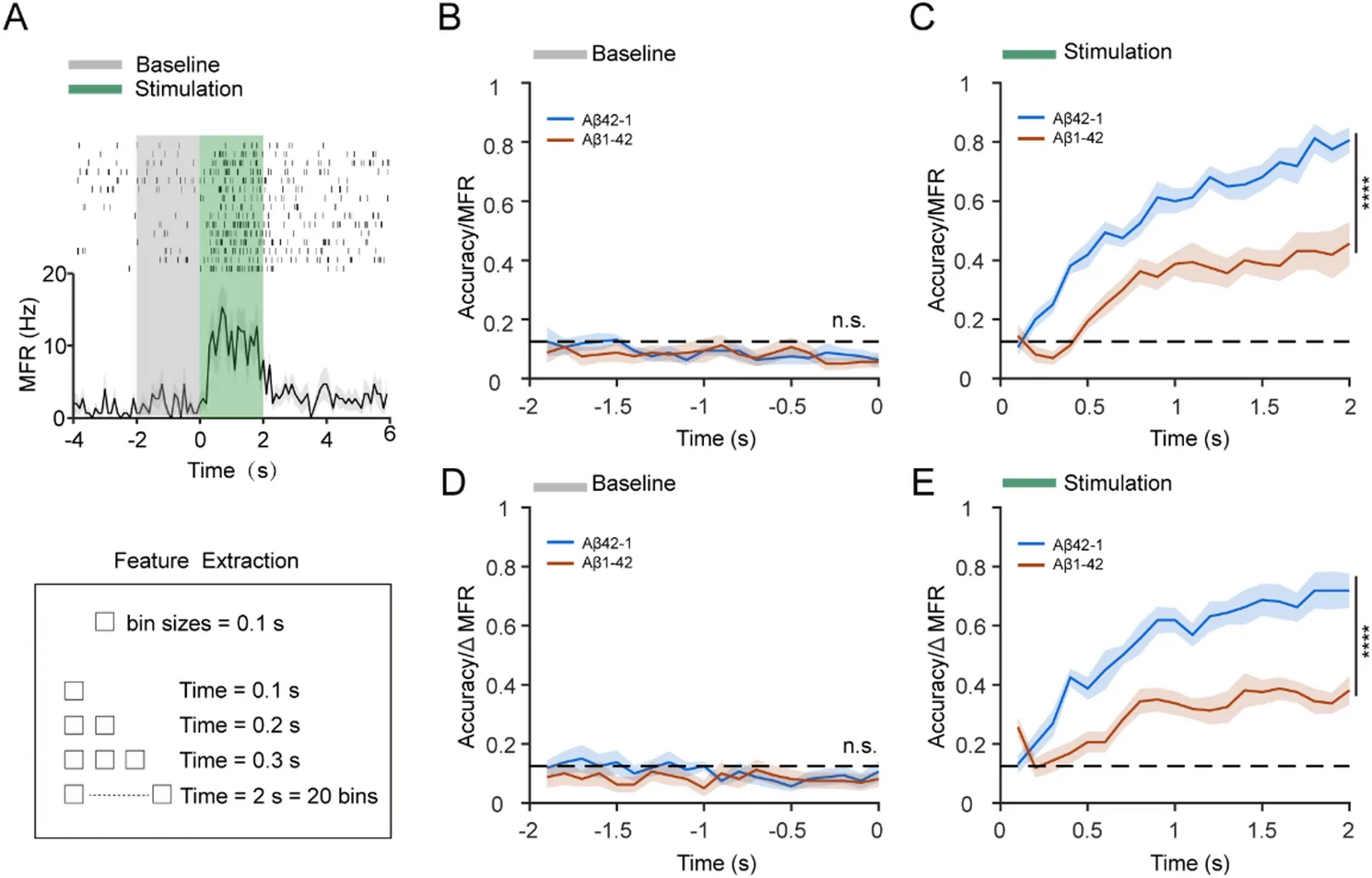
Aβ oligomers accumulation impairs population‑level odor decoding by LEC neurons. **A** Schematic of the feature‑extraction pipeline for population‑based odor decoding. Spike counts were binned into non‑overlapping 100‑ms windows to construct classification features. **B**, **D** Odor-decoding performance during the pre‑odor baseline period using MFR (**B**) or ΔMFR (**D**) features. **C**, **E** Comparison of 8-odor SVM classification accuracy between groups using MFR (**C**) or ΔMFR (**E**) features. ****p < 0.0001; n.s., not significant

Given the established role of LEC neurons in odor processing [15], we next examined whether Aβ oligomers confined to the LEC alters the ability of local neuronal populations to encode odor identity. To this end, a linear SVM with an eight-class classification framework (one class per odor) was employed to decode odor identity from population activity patterns, where chance performance was 12.5%. The feature extraction procedure and decoding pipeline is illustrated in Fig. 4A. Briefly, MFR or odor-evoked changes in firing rate (ΔMFR) of LEC neurons during the 2-s odor presentation period were used as input features. Neural activity was binned in 100-ms windows, with the cumulative number of features increasing over time (Fig. 4A). Classification performance was assessed using ten-fold cross-validation. Decoding accuracy based on odor-evoked neuronal activity was significantly reduced in mice with LEC-restricted Aβ oligomers compared with controls (Fig. 4C, E). Two-way repeated-measures ANOVA revealed a significant group effect for both MFR-based decoding (*p* = 1.05 × 10^−5^) and ΔMFR-based decoding (*p* = 4.64 × 10^−6^), indicating impaired odor classification at the population level. In contrast, decoding performance during the pre-odor baseline period did not differ between Aβ_42−1_ and Aβ_1–42_ groups and remained near chance levels (Fig. 4B, D: two-way RM ANOVA, *p* = 0.734 for MFR; *p* = 0.416 for ΔMFR), confirming that the decoding deficit was specific to odor-evoked activity rather than baseline firing patterns. Together, these results indicated that Aβ oligomers accumulation in the LEC disrupts the encoding and discriminability of odor information at the population level.

**Fig. 5:**
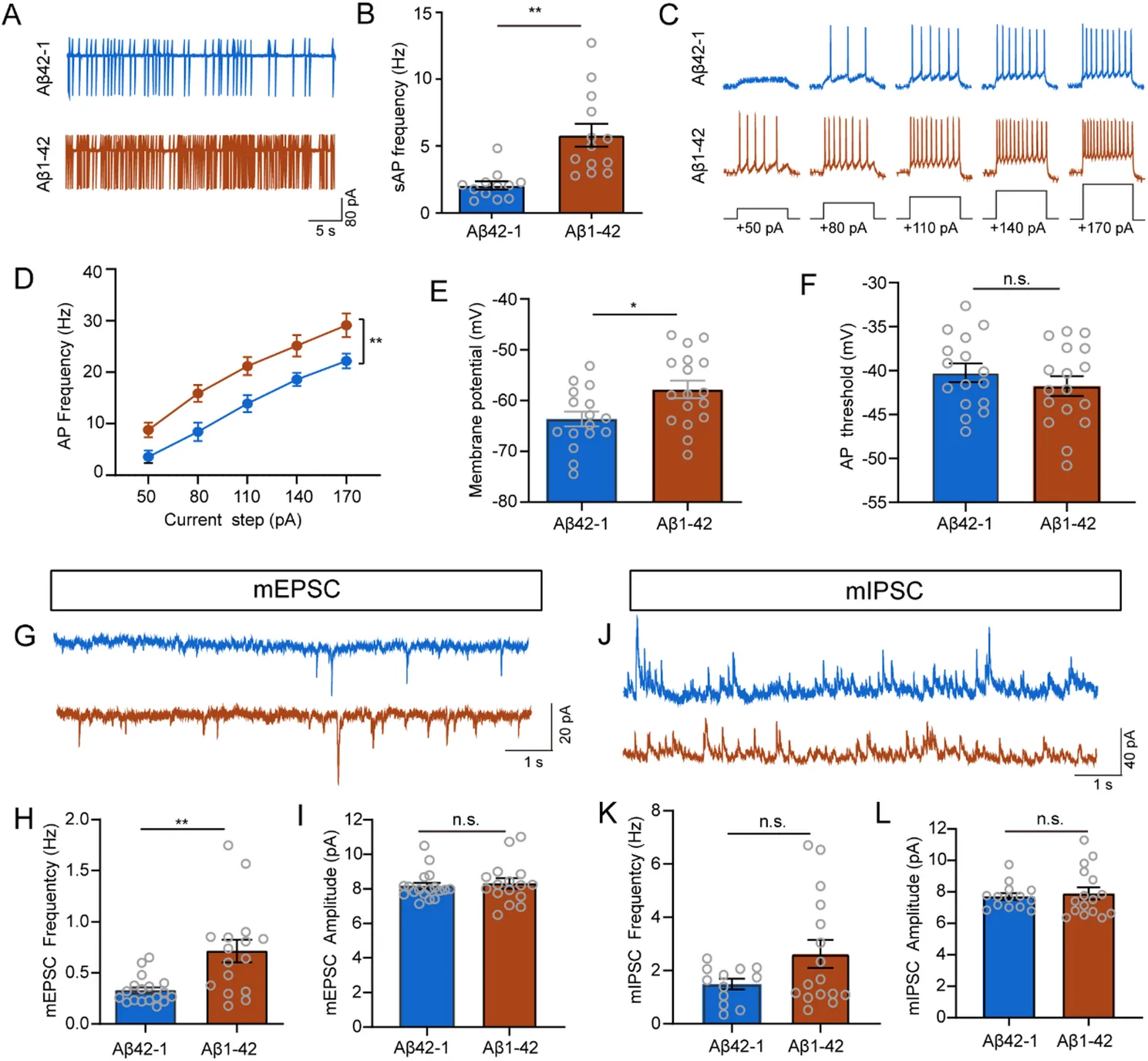
Aβ oligomers accumulation enhances excitatory synaptic transmission in the LEC. **A**, **B** Representative (**A**) and quantitative analysis (**B**) of spontaneous firing rates of LEC pyramidal neurons (Aβ_42−1_: 12 cells/5 mice; Aβ_1−42_: 13 cells/8 mice) over a period of 5 min. **C** Typical current-evoked APs in response to gradually increasing current injections in one represent pyramidal neuron from Aβ_42−1_ and Aβ_1−42_ group respectively. **D–F** Quantitative analysis of the frequency (**D**), membrane potential (**E**) elicited by positive current injections and the threshold voltage to trigger first AP (**F**) (Aβ_42−1_: 16 cells/5 mice; Aβ_1−42_: 17 cells/7 mice). **G** Representative mEPSC traces recorded from LEC pyramidal neurons in one Aβ_42‑1_ and one Aβ_1–42_ mouse. **H**, **I** Aβ oligomer accumulation increases mEPSC frequency but not amplitude. Recordings were analyzed over a 5‑minute period (Aβ_42‑1_: 18 cells/7 mice; Aβ_1–42_: 16 cells/10 mice). **J** Representative mIPSC traces from Aβ_42‑1_ and Aβ_1–42_ groups. **K**, **L** mIPSC frequency (**K**) and amplitude (**L**) are comparable between groups (Aβ_42‑1_: 12 cells/4 mice; Aβ_1–42_: 16 cells/5 mice). *p < 0.05; **p < 0.01; n.s., not significant

### Aβ oligomers accumulation potentiates the excitatory synaptic transmission in the LEC

To investigate the circuit mechanisms underlying Aβ oligomer–induced alterations in LEC neuronal activity, we performed patch-clamp recordings in acute brain slices containing the LEC. We first examined the effects of LEC-restricted Aβ oligomers on neuronal excitability by recording both spontaneous and current-evoked firing of pyramidal neurons in LEC layers II/III. In the cell-attached configuration, pyramidal neurons from both groups exhibited spontaneous action potential firing (Fig. 5A). Consistent with in vivo single-unit recordings, the frequency of the spontaneous action potential (sAP) was significantly higher in slices from Aβ_1−42_ mice compared to Aβ_42−1_ controls (Fig. 5B: nested t test, *t*_(11)_ = 3.49, *p* = 4.98 × 10⁻^3^). Next, we recorded action potentials from LEC pyramidal neurons in response to injected currents of varying intensities (50–170 pA). In both groups, action potential frequency increased monotonically with current amplitude (Fig. 5C). However, pyramidal neurons from Aβ_1−42_-treated mice exhibited significantly higher firing rates across current steps (Fig. 5D: two-way ANOVA, *F*_(1, 31)_ = 9.58, *p* = 4.15 × 10^−3^). Consistent with enhanced excitability, resting membrane potentials were significantly less hyperpolarized in neurons from Aβ_1−42_-treated mice compared with controls (Fig. 5E: nested t test, *t*_(10)_ = 2.56, *p* = 0.0282). In contrast, the voltage threshold for the first AP initiation was comparable between groups (Fig. 5F: nested t test, *t*_(10)_ = 0.884, *p* = 0.397). Together, these results indicate that Aβ_1−42_ oligomers accumulation increases the intrinsic excitability of LEC pyramidal neurons.

We next examined whether Aβ_1−42_ oligomer accumulation alters local synaptic transmission. Excitatory postsynaptic currents (EPSCs) were recorded from LEC pyramidal neurons under voltage-clamp conditions (Fig. 5G, Fig. S6A). Analysis of AMPA receptor-mediated miniature EPSCs (mEPSCs) revealed a significantly higher event frequency in Aβ_1−42_ mice compared to controls (Fig. 5H: nested t test, *t*_(15)_ = 3.31, *p* = 4.76 × 10⁻^3^), while mEPSC amplitude was unchanged (Fig. 5I: nested t test, *t*_(15)_ = 0.656, *p* = 0.522). A similar pattern was observed for the spontaneous EPSCs (sEPSCs), with an increase in event frequency (Fig. S6B: nested t test, *t*_(25)_ = 3.79, *p* = 8.55 × 10⁻^4^) but unaltered peak amplitude (Fig. S6C: nested t test, *t*_(9)_ = 4.11, *p* = 2.63 × 10⁻^3^). We next asked whether GABAergic transmission onto pyramidal neurons is affected. Miniature inhibitory postsynaptic currents (mIPSCs) were recorded (Fig. 5J), and neither their frequency (Fig. 5K: nested t test, *t*_(7)_ = 1.79, *p* = 0.116) nor amplitude (Fig. 5L: nested t test, *t*_(7)_ = 1.07, *p* = 0.320) was altered by Aβ_1–42_ oligomers. Similarly, spontaneous IPSCs (sIPSCs) showed no intergroup differences in frequency or amplitude (Fig. S6D–F). Together, these results indicate that Aβ_1–42_ oligomer accumulation in the LEC selectively potentiates excitatory synaptic transmission and intrinsic excitability of pyramidal neurons, without significantly affecting inhibitory inputs.

### Aβ oligomers accumulation in the LEC reduces dendritic complexity and alters synaptic ultrastructure

**Fig. 6:**
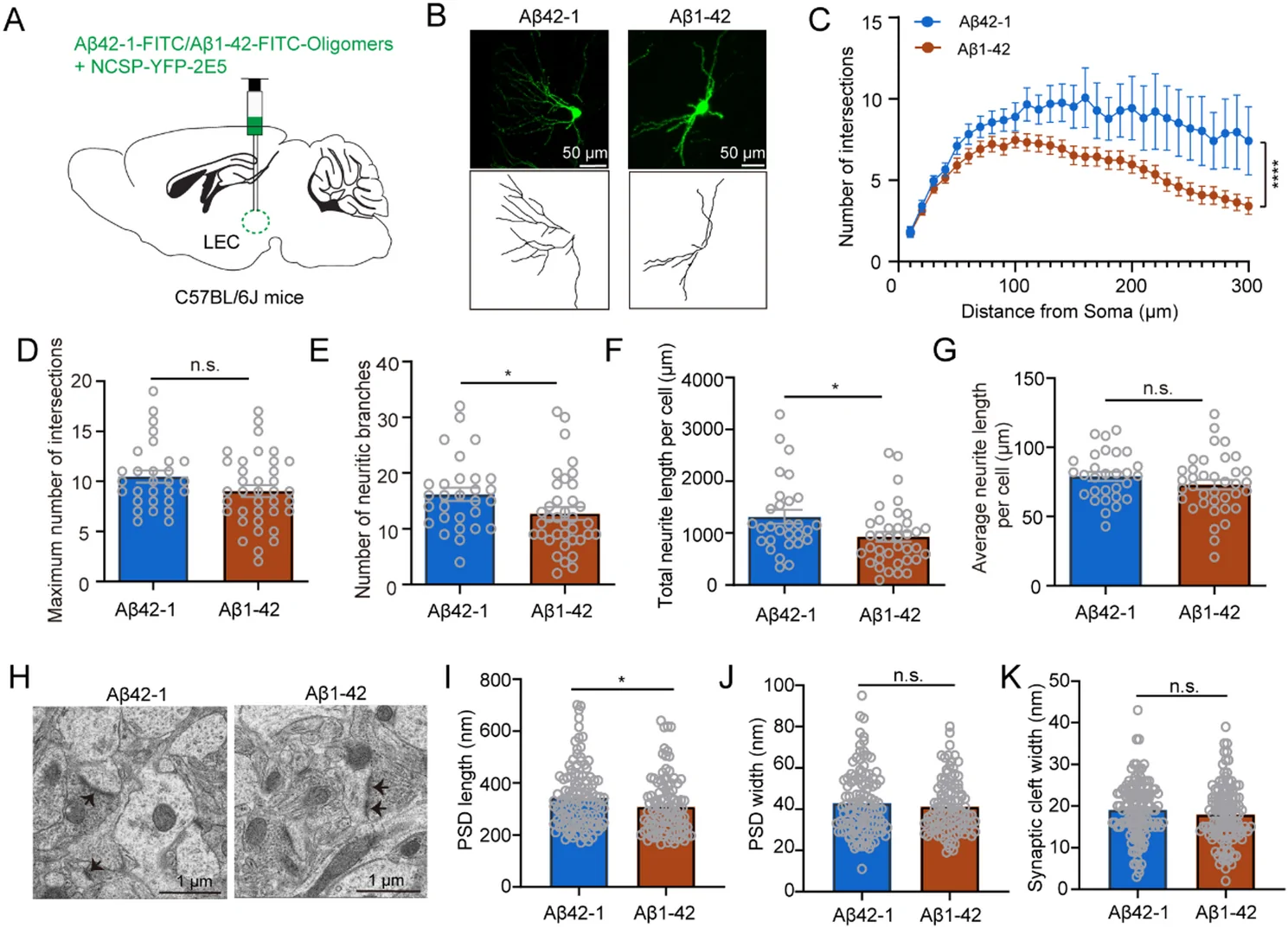
Aβ oligomers accumulation causes structural deficits in LEC neurons. **A** Schematic of the virus injection method used to sparsely label neurons. **B** Representative images showing sparsely labelled LEC neurons from Aβ_42‑1_ and Aβ_1–42_ mice. **C**, **D** Sholl analysis of dendritic complexity in LEC neurons across experimental groups. **E–G** Quantification of dendritic morphology in LEC neurons included the number of branches (**E**), total neurite length (**F**), and average neurite length (**G**). Aβ_42‑1_: *n* = 29 neurons from three mice; Aβ_1–42_: *n* = 37 neurons from three mice. **H** Representative electron micrographs showing the synaptic structure on neurons (arrowheads). **I–K** Quantification of the synaptic structure in LEC neurons included PSD length (**I**), PSD width (**J**), and synaptic cleft width (**K**). Aβ_42‑1_: *n* = 120 synapses from 3 mice; Aβ_1–42_: *n* = 113 synapses from 3 mice. *p < 0.05; ****p < 0.0001; n.s., not significant

Our results demonstrate that Aβ_1–42_ oligomers disrupt LEC neuronal activity and synaptic transmission in the LEC. To explore the underlying structural correlates, we next examined the effects of Aβ_1−42_ oligomer accumulation on dendritic morphology and synaptic ultrastructure of LEC pyramidal neurons. To assess dendritic architecture, sparse neuronal labeling was achieved by local injection of NCSP-YFP virus into the LEC of Aβ_42‑1_ and Aβ_1–42_ mice (Fig. 6A). Representative reconstructed pyramidal neurons are shown in Fig. 6B. Sholl analysis revealed a significant reduction in the number of dendritic intersections of LEC neurons in Aβ_1–42_ mice compared to controls (Fig. 6C: two-way ANOVA, *p* < 0.00001; *n* = 29 cells in Aβ_42−1_ group; *n* = 37 cells in Aβ_1−42_ group), while the maximum intersection count remained unchanged between groups (Fig. 6D: unpaired t-test, *t*_(64)_ = 1.71, *p* = 0.0913). Moreover, Aβ_1–42_ mice exhibited fewer dendritic branches (Fig. 6E: Wilcoxon rank-sum test, *p* = 0.0183) and reduced total dendritic length per neuron (Fig. 6F: Wilcoxon rank-sum test, *p* = 0.0144), with no change in average dendritic length (Fig. 6G: unpaired t-test, *t*_(64)_ = 1.23, *p* = 0.225). To further assess synaptic integrity at the ultrastructural level, we performed TEM analysis of LEC synapses (Fig. 6H). As shown in Fig. 6I‑J, the mean length of postsynaptic densities (PSD) was significantly reduced in Aβ_1–42_ mice (Fig. 6I: Wilcoxon rank-sum test, *p* = 0.0408), while PSD width (Fig. 6J: Wilcoxon rank-sum test, *p* = 0.837) and synaptic cleft width (Fig. 6K: unpaired t-test, *p* = 0.218) remained comparable to controls. Collectively, these findings indicate that Aβ_1−42_ oligomer accumulation in the LEC compromises both dendritic architecture and synaptic ultrastructure.

## Discussion

The present study identifies a causal role for the LEC in AD-related olfactory dysfunction. By selectively introducing Aβ_1−42_ oligomers into the LEC of adult mice, we demonstrate that localized Aβ accumulation impair both odor detection and discrimination. In vivo electrophysiological recordings revealed that Aβ_1–42_ oligomers elevated baseline neuronal activity and, notably, enhanced both odor-evoked responses in LEC neurons and local beta oscillatory power during odor stimulation. This hyperactive state was accompanied by impaired population-level odor decoding. Complementary in vitro electrophysiological and structural analyses further uncovered synaptic and circuit-level abnormalities that likely underlie these functional deficits. Together, our findings establish a direct link between LEC Aβ oligomer accumulation and olfactory dysfunction and provide mechanistic insight into the contribution of this region to early sensory deficits in AD.

LEC serves as a key olfactory hub within the olfactory system and plays a critical role in odor processing and associative memory [3, 13, 16]. Neuropathological studies provide the most direct and powerful evidence of early-stage pathological alterations in the entorhinal cortex of AD models, encompassing both histological changes and disruptions in neuronal activity [7, 22, 32]. However, whether LEC dysfunction directly contributes to the characteristic olfactory impairments observed in AD has remained unclear. By inducing Aβ oligomer accumulation specifically within the LEC, our study provides direct evidence that localized LEC dysfunction alone can produce deficits in odor detection and discrimination. Although the LEC is also implicated in object recognition and novelty detection [21, 29], mice in our model exhibited only mild impairments in novel object recognition. This likely reflects the strong dependence of such behavior on the integrated LEC‑hippocampal circuit, which may be severely affected during the progression of AD [22] but remained largely intact under the restricted pathology used here. These findings further underscore the specific contribution of LEC dysfunction to olfactory deficits in early AD.

One of the earliest alterations observed during AD pathogenesis is hyperexcitability in neuronal circuits, which has been proposed to originate in the LEC and propagate to other cortical regions, contributing to early network pathology [9, 25]. Therefore, our observation that LEC‑restricted Aβ oligomer accumulation elevates baseline neuronal firing (Figs. 2E and 5B) aligns with this established pathophysiological framework. Importantly, while baseline hyperactivity has been well documented, odor-evoked responses of LEC neurons under AD-related pathology have rarely been examined. Here, we demonstrate that Aβ_1–42_ accumulation in the LEC enhances both odor-evoked single-neuron responses and local beta-band oscillatory activity during odor presentation. These findings are consistent with a previous study reporting enhanced baseline and odor‑evoked LFP oscillations in transgenic AD mice [40]. The absence of altered baseline beta oscillations in our study may reflect differences in pathological burden or disease stage between models. Critically, enhanced neuronal and network responses did not translate into improved sensory encoding. Instead, population-level decoding analyses revealed a degradation of odor identity representation in the LEC following Aβ accumulation. This dissociation suggests that hyperactivity disrupts the fidelity of sensory coding, providing a mechanistic link between altered network dynamics and behavioral olfactory deficits.

Synaptic dysfunction is widely proposed as an initial insult leading to the neurodegeneration observed in AD. Consistent with this view, we observed a significant increase in the frequency of both miniature and spontaneous EPSC in LEC pyramidal neurons following Aβ_1–42_ accumulation, indicating potentiated excitatory synaptic transmission. This enhanced excitatory synaptic transmission induced by Aβ oligomers accumulation may partly explain the disturbed neural activity in the LEC. At the structural level, however, dendritic complexity and PSD length were reduced in Aβ_1–42_-treated mice. This apparent discrepancy between enhanced excitatory transmission and structural degeneration may reflect compensatory strengthening of remaining synapses following early synaptic loss. Such synaptic attenuation could itself be driven by Aβ‑induced hyperexcitability, a mechanism supported by prior studies [33]. Notably, a knock-in AD mouse model has reported a loss of parvalbumin-positive interneurons in the LEC, resulting in excitatory–inhibitory imbalance [25]. In contrast, we did not detect significant changes in inhibitory synaptic currents, possibly due to differences in pathological models or disease stages examined. These findings suggest that multiple, stage-dependent mechanisms may converge to disrupt LEC circuitry during AD progression.

Importantly, despite pronounced olfactory deficits, mice in our model did not exhibit overt cognitive impairments. This dissociation indicates that LEC-driven olfactory dysfunction can occur independently of hippocampal-dependent cognitive decline, likely reflecting an early pathological stage characterized by neuronal hyperactivity rather than cell loss. We therefore propose that progressive dysfunction of the LEC may serve as an early indicator of AD progression, preceding more widespread cognitive impairments.

Several limitations should be acknowledged. In this study, LEC pathology was modeled by local injection of Aβ_1–42_ oligomers. While Aβ oligomers are highly neurotoxic and potent initiators of plaque formation, early AD involves a broader spectrum of pathological factors, including Aβ monomers, other amyloid precursor protein fragments, and tau pathology [14, 17]. Additionally, neurofibrillary tangles are known to emerge early in the LEC [43]. Although multiple pathological factors likely converge to impair LEC function in full AD, our findings highlight that Aβ-driven LEC dysfunction alone can produce core olfactory deficits, underscoring its pivotal role in early sensory symptoms. In addition, it should be noted that although we aimed to restrict Aβ pathology to the LEC, a small amount of Aβ signal was observed beyond the LEC boundaries (Fig. 1C). Therefore, we cannot completely exclude the possibility that very limited diffusion of Aβ oligomers to adjacent areas may have contributed minimally to the observed olfactory deficits. Nevertheless, the strong predominance of pathology within the LEC supports the conclusion that LEC dysfunction plays a critical role in early olfactory impairment. Another limitation of this study is that only male mice were used. Future experiments should include female subjects to determine the generalizability of our findings.

## Conclusions

In summary, we found that localized Aβ_1–42_ oligomer accumulation in the LEC disrupts dendritic architecture and synaptic ultrastructure, alters neuronal and network dynamics, and impairs population-level odor decoding. These functional and structural changes produce AD-like olfactory deficits in mice. Given the early vulnerability of the LEC in AD, integrating measures of its structural integrity, functional activity, and associated olfactory performance may provide a promising framework for improving early disease detection and monitoring progression.

## Supplementary Information

Below is the link to the electronic supplementary material.

Supplementary Material 1

## Data Availability

All the data supporting the findings of this study are available from the corresponding author upon reasonable request.
