# Supplementary material for: Localized amyloid-β oligomers in the lateral entorhinal cortex drives olfactory dysfunction through aberrant neuronal and network activity
Source: Acta Neuropathol Commun. 2026 Jun 23;14:185. doi: 10.1186/s40478-026-02354-3 (PMC13548545; doi:10.1186/s40478-026-02354-3)
Supplement: Supplementary file 1 [file 40478_2026_2354_MOESM1_ESM.docx]

**Localized amyloid-β oligomers in the lateral entorhinal cortex drives olfactory dysfunction through aberrant neuronal and network activity**

Ting Pan^1,#^, Jiaxing Fang^1,#^, Xiao Wang^1^, Yimeng Chen^1^, Shan Li^1^, Gaoxue Qiu^1^, Changcheng Sun^1^, Chao Liu^2^, Anan Li^1,*^, Fengjiao Chen^1, *^

^1^Jiangsu Key Laboratory of Brain Disease and Bioinformation, Research Center for Biochemistry and Molecular Biology, Xuzhou Medical University, Xuzhou, China.

^2^Jiangsu Province Key Laboratory of Anesthesiology, Jiangsu Province Key Laboratory of Anesthesiology and Brain Science, NMPA Key Laboratory for Research and Evaluation of Narcotic and Psychotropic Drugs, School of Anesthesiology, Xuzhou Medical University, Xuzhou, China.

^#^These authors contributed equally.

^*^Corresponding authors: Fengjiao Chen, Address: 209th Tongshan Road, Xuzhou, Jiangsu, China, 221004; Email: fengjiaochen@xzhmu.edu.cn; Anan Li, Address: 209th Tongshan Road, Xuzhou, Jiangsu, China, 221004; Email: [anan.li@xzhmu.edu.cn](mailto:anan.li@xzhmu.edu.cn)

**Materials and methods**

**TUNEL staining**

TUNEL assays were performed by the In Situ Cell Death Detection Kit, TMR red (Roche) as described previously. Briefly, the frozen sections were rinsed twice with PBS and permeabilized in 0.1% Triton X-100 on ice for 2 min. After being washed with PBS, the sections were incubated in the prepared TUNEL reaction mixture in a dark humid chamber for 1 h at 37°C. Then the sections were rinsed with PBS again and sealed with DAPI. Brain sections treated with DNase I served as the positive control, and sections incubated without terminal deoxynucleotidyl transferase (TdT) served as the negative control. Finally, the LEC sections were observed under a confocal scanning microscope (Zeiss, LSM710).

**Behavior Testing**

***Y-maze test***

The Y-maze test was used to assess short-term working memory in mice, as previously described ^1^. The apparatus consists of three dark polyvinyl plastic arms (40 × 3 × 12 cm) arranged in a V-shape at 120° angles. During testing, mice were individually placed on the junction of the arms and allowed to explore freely over a 6-min period. The total number and the sequence of individual arm visits were recorded, and a spontaneous alternation was defined as three consecutive entries into three different arms. The alternation ratio was calculated as the number of spontaneous alternations / (the total number of arm entries − 2).

***Novel object recognition test***

The novel object recognition test was performed to evaluate cognitive function in mice based on their exploratory preference for a novel object. Before testing, mice were acclimated to the experimental room. During the training phase, two identical objects were placed in the test cage, and each mouse was allowed to explore until cumulative object exploration reached 40 seconds or for a maximum of 10 minutes. In the test phase, one of the objects from the training trial was replaced by a novel object. The time spent actively exploring each object (defined as sniffing, touching, or approaching within 3 cm) was recorded by an automated tracking software program (EthoVision XT 15). Animals that spent less than 10 s exploring both objects during the test phase were excluded. The preference for the novel object was quantified as (time exploring novel object) / (total exploration time of both objects) × 100%.

***Open field test***

The open field test was conducted in a square arena (50 × 50 × 30 cm) to evaluate general locomotor activity. Mice were allowed to freely explore the arena for 10 minutes, and their movement was monitored using EthoVision XT 15 software. To quantify general locomotor activity, the total movement time and total distance traveled in the open field were measured.

**Figures and figure legends**

**Supplementary fig. 1 Aβ oligomers are widely distributed within the LEC. (**A) Western blot analysis of Aβ_1-42_ monomer (left lane), Aβ_1-42_ oligomer preparations (middle lane) and Aβ_42-1_ preparations (right lane) using anti-FITC antibody after SDS-PAGE. **(**B) Distribution of injected Aβ_42‑1_ peptide and Aβ_1‑42_ oligomers in the LEC one week after viral delivery. (C) Representative images from three mice at 8, 11, and 15 days post‑injection (dpi), respectively, show widespread Aβ oligomer signals (green) within the LEC.

**Supplementary fig. 2 Aβ oligomers accumulated in the LEC without inducing detectable cell death. (**A, B) TUNEL staining in the LEC of Aβ_42‑1_ and Aβ_1‑42_ mice four weeks post‑injection. (C, D) DNase‑treated and TdT‑omitted samples served as positive and negative controls, respectively.

**Supplementary fig. 3 Aβ oligomer accumulation in the LEC did not impair novelty recognition or spatial working memory of mice.** (A, B) Aβ oligomer accumulation in the LEC has a minimal, non‑significant effect on novel object recognition (unpaired t test, p = 0.0674, *t*_(33)_ = 1.89; Aβ_42‑1_: n = 18 mice; Aβ_1‑42_: n = 17 mice). (C, D) Spatial working memory, assessed by the Y‑maze test, is unaffected by LEC Aβ pathology (unpaired t test, p = 0.884, *t*_(28)_ = 0.147; n = 15 mice per group). **(**E-G) General locomotor activity in the open‑field test remains comparable between groups (F: Mann-Whitney test, p = 0.305, Mann-Whitney U = 87; G: unpaired t test, p = 0.374, *t*_(28)_ = 0.903; n = 15 mice per group). n.s., not significant.

**Supplementary fig. 4 *In vivo* electrophysiology analysis. (**A) Example of spike sorting using principal-component analysis scan clustering of the extracellular voltage recorded by microelectrodes, resulting in separation of two units (unit a, red; unit b, blue). (B) Raster plots (top) and MFR (bottom) of the odor-evoked excitatory and inhibitory responses, as well as the raster plots and MFR from cell with no response to odor stimulation.

**Supplementary fig. 5 Aβ oligomers in the LEC had no significant effects on the local gamma oscillations.** (A, B) Average power spectrum (left) and the statistical analysis (right) of the ongoing low gamma (A) and high gamma (B) oscillations between groups. (C, D) Odor-evoked low gamma (C) and high gamma (D) oscillatory dynamics across all recorded mice. Normalized odor-evoked low gamma power (E, F) and high gamma power (G, H): cumulative probability (E, G) and group statistics (F, H). n = 72 odor–mouse pairs per group. n.s., not significant.

**Supplementary fig. 6 Enhanced spontaneous excitatory synaptic transmission in the LEC by Aβ oligomers.** A, representative sEPSC traces from LEC pyramidal neurons in Aβ_42‑1_ and Aβ_1‑42_ mice. B and C, Aβ oligomer accumulation elevates sEPSC frequency without affecting amplitude (5‑minute recording; Aβ_42‑1_: 13 cells from 5 mice; Aβ_1‑42_: 14 cells from 6 mice). D, example sIPSC traces in Aβ_42‑1_ and Aβ_1‑42_ groups. E and F, sIPSC frequency (E) and amplitude (F) show no significant difference between groups (Aβ_42‑1_: 12 cells from 3 mice; Aβ_1‑42_: 14 cells from 4 mice). ^***^p < 0.001; n.s., not significant.

**References**

1	Jo, K. W. *et al.* Gossypetin ameliorates 5xFAD spatial learning and memory through enhanced phagocytosis against Aβ. *Alzheimer's research & therapy* **14**, 158, doi:10.1186/s13195-022-01096-3 (2022).
